# Supplementary material for: Oscillatory movement of a dynein-microtubule complex crosslinked with DNA origami
Source: eLife. 2022 Jun 24;11:e76357. doi: 10.7554/eLife.76357 (PMC9232216; doi:10.7554/eLife.76357)
Supplement: Supplementary file 1. — The asterisk indicates the C6dT modified oligonucleotide. Small letter "tttt" indicates the four thymine (4T) linker added to prevent the aggregation of DNA rods. [file elife-76357-supp1.docx]

**Supplementary File 1.** DNA oligonucleotide sequences for the DNA origami rod. The asterisk indicates the C6dT modified oligonucleotide. Small letters "tttt" indicate the four-thymine (4T) linker added to prevent the aggregation of DNA rods.

| Seq Name | Sequence |
| --- | --- |
| Rod2_001_4T2  Rod2_002  Rod2_003  Rod2_004  Rod2_005  Rod2_006_4T2  Rod2_007  Rod2_008  Rod2_009_4T  Rod2_010_4T2  Rod2_011  Rod2_012_L  -NdT3_T30  Rod2_013_4T2  Rod2_014  Rod2_015  Rod2_016  Rod2_017_4T2  Rod2_018  Rod2_019_4T2  Rod2_020  Rod2_021  Rod2_022  Rod2_023_4T2  Rod2_024  Rod2_025  Rod2_026  Rod2_027  Rod2_028  Rod2_029  Rod2_030  Rod2_031  Rod2_032  Rod2_033  Rod2_034_4T2  Rod2_035_4T2  Rod2_036  Rod2_037  Rod2_038  Rod2_039  Rod2_040  Rod2_041_4T  Rod2_042  Rod2_043  Rod2_044_4T2  Rod2_045  Rod2_046  Rod2_047_4T2  Rod2_048  Rod2_049_4T2  Rod2_050_4T2  Rod2_051_4T  Rod2_052  Rod2_053  Rod2_054  Rod2_055  Rod2_056  Rod2_057  Rod2_058  Rod2_059  Rod2_060  Rod2_061  Rod2_062  Rod2_063  Rod2_064  Rod2_065  Rod2_066  Rod2_067  Rod2_068  Rod2_069  Rod2_070  Rod2_071  Rod2_072  Rod2_073  Rod2_074  Rod2_075  Rod2_076  Rod2_077  Rod2_078  Rod2_079  Rod2_080  Rod2_081  Rod2_082  Rod2_083  Rod2_084  Rod2_085  Rod2_086  Rod2_087  Rod2_088  Rod2_089  Rod2_090  Rod2_091  Rod2_092  Rod2_093  Rod2_094  Rod2_095  Rod2_096  Rod2_097  Rod2_098  Rod2_099  Rod2_100  Rod2_101  Rod2_102  Rod2_103  Rod2_104  Rod2_105  Rod2_106  Rod2_107  Rod2_108  Rod2_109  Rod2_110  Rod2_111  Rod2_112  Rod2_113  Rod2_114  Rod2_115  Rod2_116  Rod2_117_R-NdT3_T30  Rod2_118  Rod2_119  Rod2_120  Rod2_121  Rod2_122  Rod2_123  Rod2_124  Rod2_125  Rod2_126  Rod2_127  Rod2_128  Rod2_129  Rod2_130  Rod2_131  Rod2_132  Rod2_133  Rod2_134  Rod2_135  Rod2_136  Rod2_137  Rod2_138  Rod2_139  Rod2_140  Rod2_141  Rod2_142  Rod2_143  Rod2_144  Rod2_145  Rod2_146  Rod2_147  Rod2_148  Rod2_149  Rod2_150  Rod2_151  Rod2_152  Rod2_153  Rod2_154  Rod2_155  Rod2_156  Rod2_157  Rod2_158  Rod2_159  Rod2_160  Rod2_161  Rod2_162  Rod2_163_4T  Rod2_164_4T  Rod2_165  Rod2_166  Rod2_167_4T2  Rod2_168  Rod2_169_4T  Rod2_170  Rod2_171  Rod2_172_4T2  Rod2_173  Rod2_174  Rod2_175  Rod2_176_4T  Rod2_177  Rod2_178_4T2  Rod2_179  Rod2_180  Rod2_181  Rod2_182  Rod2_183  Rod2_184  Rod2_185  Rod2_186_4T2  Rod2_187  Rod2_188  Rod2_189  Rod2_190  Rod2_191  Rod2_192_4T2  Rod2_193  Rod2_194  Rod2_195  Rod2_196_4T2  Rod2_197  Rod2_198  Rod2_199  Rod2_200_4T  Rod2_201_4T2  Rod2_202  Rod2_203_4T2  Rod2_204  Rod2_205  Rod2_206_4T  Rod2_207  Rod2_208_4T2  Rod2_209_4T2 | ttttGGGCGATGGCCGTCTATCAtttt  CCAACGTCGTAATCGCGTGCCTGTTCTTCTCGTCGGAATAAG  AAAGAACGTGGACTCAAATCAAGTTTTTACTATCGATTTTGAGATTAGA  CACTATTTTCCTGTACCGGGGGTTTCTGCCAGAGCACATCCT  AAGAGTCGAGGTGCCGTAAAGTGCCTGAAAATGGA  ttttTTGAGGATCCCCGGGTGTGAGCCTCCTCACAGtttt  ACCGAGCTCGAATTCAAAGGGCTTGCAGCAACAAC  GGCGCTTTCCTTAGTGACCTCCGGCCAGCACATGGTCATAGCTGT  ttttCGACAGTATCGGCCTCAGGAAGAAAACGATTTTTCGTCGCGTCC  ttttGCAAACGCGGAAACACCAGAAtttt  CTGGCAGTGAAGGGTAAAGTTTCGCACTGCCCGAA  *T*T*ttttttttttttttttttttttttttttttAGCCGCACAGGCGGCCTGGCACCGCTTCTGGGTCACGTTGGTGTAGATG  ttttATATATTCGGTCCTGACGAGTCCGTTGCTGATTGCCGTTCCGtttt  CATAACGCGGTCATGTGAAATTGTTATCTTGGAAC  CAACAGGCAACGCAGTCAAATCACCATCGGGATAGTGCCGGAAAAA  CCAGCCAGCATCGTAACCGTGTTCAAAA  ttttAGTAATGTGTAGGTAAAGACATCTGCCAGTTTGAGGGGACGAtttt  GGAGACAAGGATAAAAATTTTTAGAACCGGGTGAGAAAGGCC  ttttGTTTTAATTATGCAATGCCTGtttt  CTCATATATTTTAACGAGCTTAGACTTCGAGATGG  CAAAGCGGCAAGGCCTGAACAATAAAAACAGGGAACTAATGCAGAACGC  AACCAGACCGGAAGAAGATTATAATCATGCTGCTC  ttttCGAGTAGTAAATTGGGCTTAAATATCGCtttt  GTTTGCCGACTCCTCAAGAGATCAACTTAGAGGAA  TTTAATTAGGATTAAGCGACAGTCCAGACGACGACTCGCTAT  TGTGAATAGGCTGATTTAGCGGACAAAAAATAGATAAGTCCT  TACCTTACGGATTGCATCAAACAAACTCCCAGTAGTTGAGCG  GCTTGCCGCTGAGGCGAAAAACCCACTACGTGAACATCCAGAAACGCTC  ATTAAATGCCGTCAATAGATAATGGAAAACAATGAGGAGTTAATTCAGT  ACTAACAACTAATACGCTCAATTGATACGGGATCGATCAACGTAACAAA  AGAAGCCTTTATTTTCAGGATAGCAAAGTGCGATTTACCCAA  AAGGCCGCTTTTGCCGATAGTCCGGAATAAACAGT  TCACCCTCAGCAGCATTTCTTACTCAGGTTGAATT  ttttCCATTGCAACACGCATAACCGtttt  ttttACCAGGCGGATAAGTGCGGGGTTTTGCTCAGTtttt  CCGTCGACATCGCCCAGGAAAACAATATTACCGCCAG  GAGGGTTATGTGAGTAATTAATTTTCCCTAATTCTGAATCAAACCATTA  ATACATTATTCGACAACTCGTTCAATATGATATAA  GTATAGCTGCGCCGTACCTACGCCTTGCTGGTAATCATCACC  TCACCGTAAACAGCTCGTCTGGTAGAAGAACTCAATGGGGTC  ttttGGAGAATTAACTGAACACCCGGAAACAATCAGTGGATTAG  CCTTTGCCCGAACGTTAATGGAGGTGTATATTAAG  AAATAGCCTAATATCAGAGAGAGCCAGCGTAGCGCATGAAAG  ttttTCGATAGCAGCACCGTGTCACCAATGAAACCAtttt  GCCTGTTGAATAACAAGTCAGAGGGTAACACCATT  CCTTGAAAACATAGAAGTACCTCAGACTAAAATCA  ttttCATGTTCAGGCGCATTAGACGtttt  AGCCTTTACAGAGATATCAACGGTAAAGTTAGAATACATAAA  ttttTGTAAATCGAATAAACAAtttt  ttttTTAGACTTTCCTTGCTTCtttt  TGAATAAACAAACATGAGGATTTAGAAGTAtttt  AACGTCAAAAATGAGAACAAGGAATATACGATAGCACCTTTTTTATTAA  ACCCTGAATTATACTGACAAGAGCATCGAGTGTTGTTCCAGT  ATATGTAGAAGAATGCGAATTCCACACAACAAGGGTTG  CCGAGATTACGAGCTGGTGCTGCGGCCACGTCAGCGTAATCT  GAATAGCCCGATTTAGAGCTTAACCGTTAGTAATA  CAACTGTCAGTTGGTGGTCTGCACTCTGCGGAAGCATAAAGTATCAAAA  TTCAGAAAAAACGAGACCAGGACTAAAGAAAATCCCTTATAA  TGCCAAGAATCAGTGTCCTGGGGTGCCTAATAATCGGC  CGCTCACGCGGGCCGTTTTCAGAACGTGTATTCATTTAAGAA  GTAAAGCACTGCGCGCCTGTGGTCAGCAGCTGGCT  GTTCCGAGAGTGAGCAGACGATCCAGCGGCCAACG  ATCAAGAGTGGTGCGCGGTTGTGTACATCGAC  ATGAACGGCAGCACGGATCAAACTTAAATTTC  AACCGGACCGGACTAAAAAATCCCGTAAAACCAGG  TACCTTTTTGCGGGTATTCAACCGTTCTAAACGGCCCATTCGCCATTCA  CGCATAGGCAACCGTCATTTGCCGCCAGTGGGAAG  TGGCTTACTAAATCGCTATTTTTGAGAGAAATGTGGGTGCGGGCCTCTT  GACGTTGAAAATCAGGCTGCGTTCTCCGTGGGAAC  CCCTTATATCACCGATAAGAGCATTATGAAATTAATGCCGGACGTCGGA  CTGGCTCCTATTATCAAAGCGGGATTGACCGTAATAATATGA  AGCTGATACCCTGTAATACTTAATTGCT  CGTTAATAACGAGAGGCGATCAGCGAGTAACAACCGAGGGTA  GGTTGTACCAAAAAGTCATTTTAAATCAGGAAGAAGACCTTCACGGCTA  CGCTATTCTTTCATCAACATTATCTACACTCAGAGCATAAAGGAGCTTA  AGTCAGATAGAGAGGAATTAGATAACCC  GGTCTTTCCTTTTGTCACCGAAAGCCCAATAAGAAACGATTTATCCTAA  ATGACCATTGCGGAATTAAAGAAACAAT  CAGTCAGCCTATTTCATAGCCCAGAGGCTTTACGAGCATGTAAATCCAA  AAATCTAATAAACATGCCATCAACGCCAATCAATAATCGGCT  GTGTACAACTAACGGCCTTGAATCACCGTTGAGAACTTATCATTCCAAG  ATCTAAAATATCTTTTATTTAGAGGTGAGAAAGAC  GAACGAGATCAGCTCACCCTCAATTACAAGTCAATAGTGAATATTTAGG  TGAAAGGAATTGAGCCAGTCATCGGTTTGGTAGCA  CAGAGGCAGCCTTTCAGAACCAAACATC  GTCAGTTGGCAAATAAAGGGACAAAAGGTTTGAGG  ACTTTTTCAAAAAACACCACCAGCAAAACCTTTTTAACCTCCGGCTTAA  GTACCGCTGCTTTCCATTGGCGTAATAACATCACTCACTAAA  CCACCCTAATTGTACACGACCGTAGCAATACTTCTGGAGCCC  TCAGAGCAAGGCTCCATTCTGCCATCACGCAAATTGACGGGG  CTGAAACGTTTTCAAATAAGAAAAAATAATATCCC  TAGGAGCTTTTAAAAGTTTGAATTTCATAGGTTTATATTATT  TTTGTTTACAAGAATTGAGTTCTTGAGCTTTCGGTCGGAACC  GAAGGTTTATCATTTTGCGGACAAAATTAGAACCGCCCCCTG  ATAATAAGAGCAAGGTGAATTTAGCGTTGTTAATG  CAACAGTAACCACCAGAAGGAATGAAACGCCACCCTGCCCGT  AACAGCCGAAATAGCAATAGCGACGGAAAATCAAAGTAACAG  TTAGATTAAGACGCAGCCAGTTCGGCATCATTTGG  AATCATAGGTCTGAATTTAACTTTTCATATTATTC  AATGCTTTAAACAGATTGCTGAAATATTTATCTTA  ATATTATTTATCCCGAAACCAACATGTATTATCAAAAGAAAAACAAAGA  CCAGTTACAAAATAGTCTTTCTCGCCATGAGACTAGAAGATGGCGGAAT  ATTTTCGTGAGAAGTTTAACAGTAACAT  TATCATCATATTCCTTACCTGCTCATTTGGTCAGTGAACAAC  AAAGCCGGCGAACGAGTCTGTGCCAACAATATCTG  TCGGAACCCTAAAGTTGATTAAGATTCA  GATGGTGAAAGGAAGGGAAGAAGTGAGGCTGACCT  ACGCCAGAAACAGCCGTCGGTCCTGCATCTAACTCACATTAACCTGTTT  CAGCAGGCGAAAATTTGCGTTCGGTGCCGGTGCCCGGTGCCA  ATGTGCTCTCACGGCAACCAGCAGCCAGGCGCTCACTGCCCGTTTGCCC  ACCACATGACGGTCGGCACCAGCGGTCCACGCTGG  TTGGGTACTCCGTGTGTCCAGCATCAGAGTCGGGAAACCTGTGCAGCAA  GAGAGTTCGTGCCACAAATCGTTAACGGCATCAGCGGGGTCA  CTTTCCATGCCGGGTTACCTGCTTACGG  TTGCAGGTGTTCAGGCTGCATTAATGAA  ACCAACTTCCCACGAAAAAGAGACGCAGCTGGCGA  *T*T*ttttttttttttttttttttttttttttttTTGTGAGAGATAGACTTTACGCCAGGGTTTTCCTCATTTTTTAACC  AATCATACTGGAGGGTGAAGGGATAGCTGCAAGGC  GAACCAGGGAAGGTAATATAAATAAAGCAAGGCTATCAGGTCTAGCCAG  CCGCCTCACATTCAATGTTTTAAGAATTGAGAGTCTGGAGCAAATTCGC  CCCTCAGAGCGCCAAGTACGGATAAATCGAATCGATGAACGGAATAGGA  AAAGATTATATTCAAAGGGGGGTCTGGCCTTCCTGATTGCCT  TAGGAATAATACTGGATTAAGACGCCATCAAAAATAACAAGA  TAATCGTAAAACTAGCATGTCAAATCAGCCAGTCACGACGTT  AGCAAAATTAAGCATGCTGTACCCCTCAATTATTAAGGACAGAAGTTTC  ATACAGGCAAGGCAAAATATGGTCATAACATCAGTCGAACTGAATACGT  AGCATTAACATCCATGTCTGGAGCGTCC  GAGCCACAGAAAATATTCCATTAGTAGT  TTGAATCGCTCAACACCGATTAAGAAAA  CGGAATCCAACTAAAAGACAAGAACAAA  ATGCAGATGTTTAGACTGGATAAGTTTCTCATATGACCGAGG  TTGAAAGCAGGTAGATAAGTTACCGGAAACGCTCAATTAAACCAAGTAC  AGGGAACTGAGATTTGATACAGCCGCCAATTCTTAATCGAGAACAAGCA  AGGCGCATCAACTAAGCGTCAACCCTCAAGTATCATTTATTTTCATCGT  GCCACGCTGAGAGCCTATTAGCAGCGGAACGAAAGCGGAACG  CAAACCCTCAATCAGAGATAGAAATCTCCATGAGG  CATTAAATTTTTTCTAGCAAGATTATTCGTTGGGTTATATAAAAAGCCA  ACCTTGCTGAACCTGAAAGCGATAATAACGGGTAA  AATGCCATAAAGGAATGTACCTACAAAATAAATGCTGATGCATATACAA  AAATGAAAAATCTAACAGACAGAACAACCTACGAA  ACCTAAAGTGAGAACGTCACCTGATTGCTCGCAAGACAAAGACCTGTTT  TCAGGGAACGTTGAAACCCTTCCACCGAGTAAAAGTGGCGAG  GGAACCCATTGCGATAAGAATGTGTTTTTATAATCAAGCGAA  TGAGTTTTAGAAAGATATTTTGGTACGCCAGAATCGGGCGCT  ACTACAAACAGTTTTCTTTAAAAAGGGATTTTAGATCACGCT  TAATTTGCCGAAGCCCTTTTTGAGGGAGAGCCACCTTAACGG  CAAATATCAGATGATGGCAATGAGGCGACCCAATAACTGGTA  AACGAGCGTAAGCAGATAGCCAAGGGCGCCTCAGAGGAGTGT  AAGCATCTATAATCCTGATTGACCAAGTGTAACACCTTTTGA  ATCCTGAGTTACCAGAAGGAAGTTTACCAACCGCCTACATGG  CAGCAGCTATACTTCTGAATAATTCGCCAGTACAATCCAGTA  TTTGCACAAACGCAATAATAAAATCAATCACCCTCTTACCGT  GTCTTTCCAGAGCCAACGGGTACAGTAGGGCTTAGATTTCAATGATTAT  ATCTTACCAACGCTCGCACTCCCAGTATCTATATGTCGCGCATCATCAA  CCAGCTACAATTTTAGCCGTTTATGCGTAATCCAATTTGAATTTTGGAT  AGGAGCGGGCGCTACTGAGAAACGTGGC  GGCAAGTGTAGCGGCAGGAACTGAATGG  CCGCCTGGCCCTGAGCGCGTAACCACCAGCCGATTTGCGCGA  CCCTTCATCGGCCACCCTTACACTGGTGCGCTTTC  AGTTTTGACGAGGCATCCGCGCACTCATCTGATTG  GACGGCCCGCCATGATCCGCCTAAACATACGCGCGGGGAGAGGCAACAG  GAGACGGGCGGTTTTTCTTTGCTCGTCAGGGCGCG  CACCAGTGGGCGCGTACTATGCGTGCTTGAACGAACCACCtttt  ttttGGTTTTTCTTTTCCAAGCGGACGTTAGATCTAA  AACAATCTATGAGCTAAAGGTGCGTATTGGGCGCCAGGGT  TGCGGCTGGTAATGGGCGGGTCAGTATCATTAACCCT  ttttCAACGGAGATTTCTGTTGCCCtttt  ACCTGCTGCACTCATTTACCAGTCCCGGAAT  ttttGGATAACCTCACCGGAGAGCCGCCAAAATATAGATAC  CCCCAGCTAAATTGGTTGCGGGGCGAAACGTACAGAGTGCCA  CATTTGGAAAAGCCCCAAAAATAAACGTAGAGGTG  ttttGACGATAAAAACCACGGGAACtttt  CCACCAGGTTTATTCCAATTCAGGTGGCTGTACCCCGGTTGAAATTCGC  TAATCAGGGCGCGAGCTGAAATGCGAAC  GCTTTTGAGCTTTCTAATATTTTGTTAA  ttttGCAAATATTTAAATTGCAGGAAG  AGGAATTCCAGAGGGTAAAACATTAAATTTTTGTTAATCATA  ttttGTCAATAACCTGATTGTATAAtttt  ATCAATTCTACTAAATAACAGAGTAAAATACATAAACTTAGCAGGCAAA  TTTAGCTATATTTTTGACCATGCGAGAGACTATCACGCCTGAGATTATA  TAGTTAATTTCATCTTAAATACCGCCGCCGCAAAGTTTAGTT  ATACCGACCGTGGGTTGAGGGTGGCAATTTCGCAAATG  GGGTAATTTGATTCTTGTCACCGGAATAAGATTAG  CAAAAGAGAGTAGAACACCACCATGATT  TGCACGTATACAGTATAGTTACATTAAAGAGCAAC  ttttATTGGCCTTGATATTCACACGTTTACCAGACtttt  CCATGTTCGCCAAATCTGAATAGAGCCGATTACTAATTACCGCGCCCAA  TGTCGAAATAGTAAGCCAGAAACCAGAGAGAATAACAAATCAGATATAG  AGAATACACTTTCACGCCTGTAGAAACAAAAACTTTTTCAAAAATCATA  ATTAACACCGCCTGACTGATACTAAACAACTAAAA  CTTTGACTGTATGGAGCCCTCAACAGTA  ttttGTCTTTCCACGAAACAAAGTAtttt  TTCTGACGATGAATAAAACAGAACAGAGGTGAGGCCCATTAAAATTTTC  CACAGACGATTTTGGCCCTAAGAGCTAAACAGGAGCACCCGC  GCGTAACGTAAATGAAATACCTCCTCGTTAGAATCCGCTACA  ttttATTTTCAGGTTTAGTTTTGTCtttt  CAACAGTGGTTAGAACCTACCCATCGGGAGCATTCCGCAGTC  TAAATCACCCAAAAGAACTGGGGAATAAAACCACCTGGAAAG  TTGCGGGAAGACTCCTTATTAAAAGAAACAGCATTAAATCCT  AACAAATGACAGGATGATAAAATCCGGTATTCTAAGAACGtttt  ttttTACATACATAAAGCAGGTCAGACGtttt  ACATATACGCAGTAGCGAACCTCCCGACAAGGCTTTAAGGCG  ttttCGAGGCGTTTTATGTTAGCAAACGTAGAAAAtttt  TTGCTATAGGAATCGAAAAAGACGCGAGATAACGGATGGAAG  AGGTTTTGAAGCCTTAGCAAGACACCGGTATATTTCCTTTTAATATCAA  AACGTCACTAAATTTAATGGTTTGAAtttt  CGCGCTTAATGCGCAGAGCGGAACATCGGGTCAGTAATTATT  ttttAGCAGAAGATAAAAATAAAGAAATTGCGTAGtttt  ttttACGAGCACGTATAAGTTGCTTTGtttt |
